# Multi-omics HeCaToS dataset of repeated dose toxicity for cardiotoxic & hepatotoxic compounds

**DOI:** 10.1038/s41597-022-01825-1

**Published:** 2022-11-14

**Authors:** Marcha Verheijen, Ugis Sarkans, Witold Wolski, Danyel Jennen, Florian Caiment, Jos Kleinjans, HeCaToS Consortium, Irina Agarkova, Francis L. Atkinson, Ivo Bachmann, Vanessa Baier, Gal Barel, Chris Bauer, Twan van den Beucken, Stefan Boerno, Nicolas Bosc, Conn Carey, José V. Castell, Olivia Clayton, Henrik Cordes, Sally Deeb, Hans Gmuender, Stefano Gotta, Patrick Guye, Anne Hersey, Ralf Herwig, Stephane Heymans, Peter Hunt, Fiona M. I. Hunter, James Hynes, Hector Keun, Eirini Kouloura, Lars Kuepfer, Laura Kunz, Alex Lewalle, Matthias Lienhard, Teresa Martínez-Sena, Jort Merken, Jasmine Minguet, Nhan Nguyen, Steven Niederer, Ramona Nudischer, Juan Ochoteco Asensio, Bernardo Oliveira, Christian Panse, Carla Pluess, Adrian B. Roth, Ralph Schlapbach, Yannick Schrooders, Johannes Schuchhardt, Matthew Segall, Nathalie Selevsek, Pilar Sepulveda, Ines Smit, Christoph Thiel, Bernd Timmermann, Timo Wittenberger, Alexandra Zerck

**Affiliations:** 1 Department of Toxicogenomics, GROW - School for Oncology and Reproduction, Maastricht University, Maastricht, The Netherlands; 2 European Molecular Biology Laboratory, European Bioinformatics Institute (EMBL-EBI), Hinxton, UK; 3 Functional Genomics Center, ETH Zurich, Zurich, Switzerland; 4 Insphero AG, Schlieren, Switzerland; 5 MicroDiscovery GmbH, Berlin, Germany; 6 Institute of Applied Microbiology, RWTH, Aachen, Germany; 7 Department of Computational Molecular Biology, Max-Planck-Institute for Molecular Genetics, Berlin, Germany; 8 Max-Planck-Institute for Molecular Genetics, Sequencing Unit, Berlin, Germany; 9 Luxcel Biosciences, BioInnovation Centre, UCC, Cork, Ireland; 10 Experimental Hepatology Unit, IIS Hospital La Fe, Valencia, Spain; 11 Roche Pharma Research and Early Development, Roche Innovation Center Basel, Basel, Switzerland; 12 Genedata AG, Basel, Switzerland; 13 CARIM School for Cardiovascular Diseases, Maastricht University, Maastricht, The Netherlands; 14 Optibrium Ltd., Cambridge Innovation Park, Cambridge, UK; 15 Cancer Metabolism and Systems Toxicology Group, Imperial College, London, UK; 16 Department of Biomedical Engineering, King’s College London, London, UK

**Keywords:** Toxicology, Experimental models of disease

## Abstract

The data currently described was generated within the EU/FP7 HeCaToS project (**He**patic and **Ca**rdiac **To**xicity **S**ystems modeling). The project aimed to develop an *in silico* prediction system to contribute to drug safety assessment for humans. For this purpose, multi-omics data of repeated dose toxicity were obtained for 10 hepatotoxic and 10 cardiotoxic compounds. Most data were gained from *in vitro* experiments in which 3D microtissues (either hepatic or cardiac) were exposed to a therapeutic (physiologically relevant concentrations calculated through PBPK-modeling) or a toxic dosing profile (IC20 after 7 days). Exposures lasted for 14 days and samples were obtained at 7 time points (therapeutic doses: 2-8-24-72-168-240-336 h; toxic doses 0-2-8-24-72-168-240 h). Transcriptomics (RNA sequencing & microRNA sequencing), proteomics (LC-MS), epigenomics (MeDIP sequencing) and metabolomics (LC-MS & NMR) data were obtained from these samples. Furthermore, functional endpoints (ATP content, Caspase3/7 and O2 consumption) were measured in exposed microtissues. Additionally, multi-omics data from human biopsies from patients are available. This data is now being released to the scientific community through the BioStudies data repository (https://www.ebi.ac.uk/biostudies/).

## Background & Summary

The main goal of the EU/FP7 HeCaToS project (**He**patic and **Ca**rdiac **To**xicity **S**ystems modeling) was to aid predictive human safety assessment using alternative approaches to animal testing. The project focused on assessing toxic cellular responses in liver and heart. We emphasized on these organs because they represent the primary target for repeated dose toxicity in drug-treated humans.

In order to obtain a mechanistic understanding of toxicological responses in these target organs, we generated *in vitro* multi-omics molecular data obtained from innovative 3D human hepatic and cardiac microtissues upon perturbation by toxicants. DNA, RNA and proteins were isolated from these microtissues for analysis with epigenomics, transcriptomics and proteomics techniques respectively. Furthermore, media of exposed microtissues were used for metabolomics analysis. Additionally, some functional measurements (ATP content, Caspase3/7 and mitochondrial O2 consumption) were performed.

**Fig. 1:**
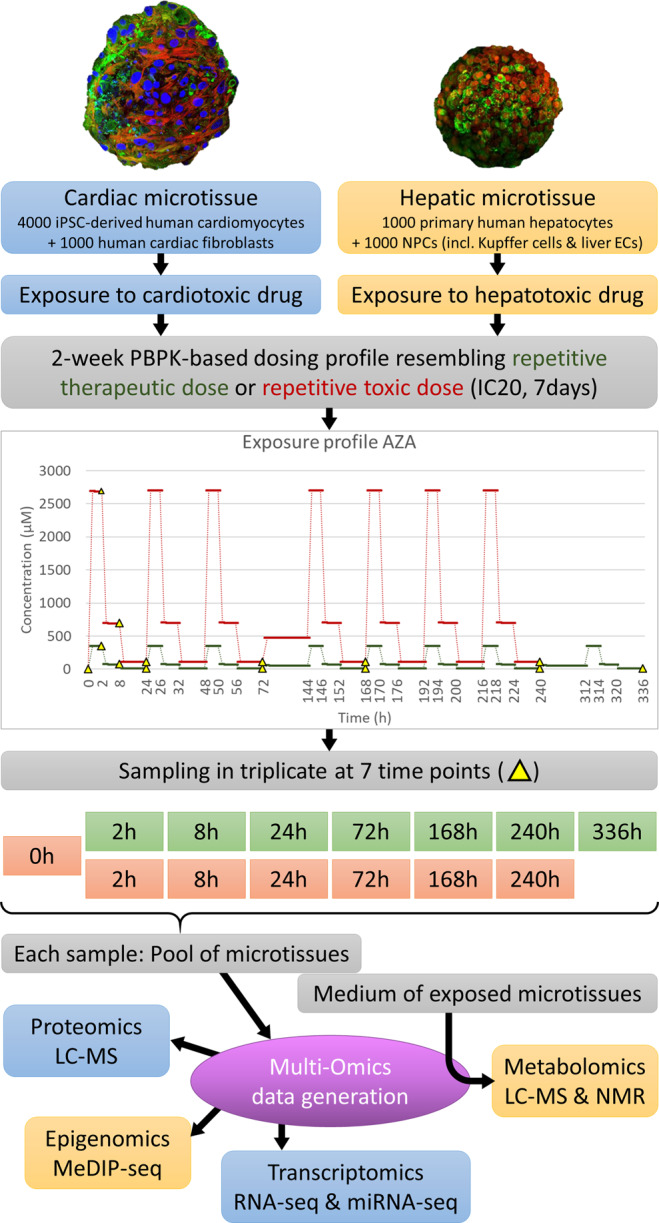
Overview of experimental design. The *in vitro* experimental design of the HeCaToS project made use of 3D cell models called microtissues for the assessment of repeated dose toxicity. PBPK-modeling was used to obtain dosing profiles resembling the repetitive administration (1x daily) of either a therapeutic or a toxic dose of the investigated compound. In the experimental design, this dosing profile was realized through three medium changes per workday (high dose for 2 hours, medium dose for 6 hours and low dose for 16 hours). During weekends (72h–144h and 240h–312h) the microtissues were exposed to a calculated average concentration without medium refreshment. The 14-day exposure profile included 7 sampling time points: 2-8-24-72-168-240-336 h for therapeutic doses (green) and 0-2-8-24-72-168-240 h for toxic doses (orange), though the 0 h baseline sample can be applied for both doses. For each sample, multiple microtissues were pooled before isolation of DNA, RNA and proteins, which were analyzed with epigenomics, transcriptomics and proteomics techniques respectively. The medium of the exposed microtissues was used for metabolomics analysis.

This goldmine of information^1^ was generated for 10 hepatotoxic and 10 cardiotoxic compounds (Table 1). Repeated dose toxicity was investigated for each compound by exposing the *in vitro* 3D microtissues (either hepatic or cardiac) to physiologically relevant concentrations or a toxic dosing profile for 14 days. PBPK-modeling was used to mimic the intraday fluctuating drug concentrations in the target organ thus resembling a patient receiving a single dose per day, which were experimentally incorporated by changing the medium three times per workday^2^. To monitor the molecular changes over time, samples were obtained at multiple time points; 0h-2h-8h-24h-72h-168h-240h-336h. Figure 1 presents a visual overview of this general experimental design.

**Table 1:** Hepatotoxic and Cardiotoxic compounds.

Hepatotoxic compounds(abbreviation)	Drug class	Cardiotoxic compounds (abbreviation)	Drug class
Fluorouracil (5FU)	antimetabolites	Fluorouracil (5FU)	Antimetabolites
Acetaminophen (APAP)	analgesics and antipyretics	Doxorubicin (DOX)	Anthracyclines
Azathioprine (AZA)	immunosuppressants	Epirubicin (EPI)	Anthracyclines
Cyclosporin A (CYC)	immunosuppressants	Idarubicin (IDA)	Anthracyclines
Diclofenac (DIC)	NSAIDs	Daunorubicin (DAU)	Anthracyclines
Isoniazid (ISO)	antituberculosis agents	Amiodarone (AMI)	Antiarrhythmics
Methotrexate (MTX)	antimetabolites	Celecoxib (CEL)	NSAIDs, COX-2 inhibitors
Phenytoin (PHE)	anticonvulsants	Docetaxel (DOC)	Taxanes
Rifampicin (RIF)	antimycobacterials	Mitoxantrone (MIT/MXT)	Anthracenediones
Valproic acid (VPA)	anticonvulsants	Paclitaxel (PAC/PTX)	antimicrotubule agents

Drug classes were obtained from MedlinePlus. U.S. National Library of Medicine^44^.

Some of the data generated in the HeCaToS project have already been published^2–14^.The best reflection of the use of HeCaToS multi-omics data is contained in a paper entitled: Network integration and modeling of dynamic drug responses at multi-omics levels^2^. This paper provided proof-of-concept that the data generated in the HeCaToS project are suitable for obtaining an integrative understanding of dynamic adverse drug responses across dose and time. The application of multiple high-throughput omics technologies provided a complete view of the molecular responses that resulted from anthracycline exposure (Doxorubicin, Epirubicin, Idarubicin and Daunorubicin) of the cardiac 3D microtissues. Dynamic changes over time were observed for 641 proteins and 904 genes. By integrating this data into a protein-protein interaction network (see original publication^2^), we identified a mechanistic network containing 175 proteins that formed a common signature for the anthracyclines. Furthermore, 70% of these response proteins could be validated against omics data generated from human cardiac biopsies (taken from dilated cardiomyopathy patients with and without historic anthracycline treatment).

Additionally, to unraveling biological effects, the HeCaToS data has also been used for the development of improved bioinformatics approaches. For instance, the bioinformatics tool FuSe^15^ improves transcriptomics analysis by grouping transcripts with similar function as obtained from Acetaminophen (APAP)-exposed liver microtissues, rather than grouping all transcripts (coding and non-coding) for a specific gene. Furthermore, the HeCaToS data was used to develop an omics data analysis framework for regulatory application (called R-ODAF) for transcriptomics data^16^. This user-friendly pipeline covers all the required elements of a complete data analysis, from data quality control, outlier removal, and raw data conversion to process read counts to the final statistical analysis to identify differentially expressed genes (DEGs). Furthermore, the R-ODAF was optimized to increase biological relevance to allow proper regulatory evaluation of risk assessment. To this end, additional statistical filtering steps (stringent DEG criteria and filtering for technical spurious spikes) were added to remove false positive DEGs that may influence biological conclusions drawn from the data.

Still, most of the data has not been explored to the detail it deserves. Therefore, we are now releasing the data (for an overview see Appendix I) to the scientific community to enable other researchers to benefit from the data, extend the collective scientific knowledge and facilitate future scientific discoveries. As a courtesy, we ask researchers who make use of the data, to cite this data release paper. All data has been stored in the BioStudies data repository^17^ (https://www.ebi.ac.uk/biostudies/).

## Methods

### 3D microtissues

The 3D InSight^TM^ Human Liver Microtissue is a commercially available model that consists of approximately 1000 primary human hepatocytes in co-culture with approximately 1000 non-parenchymal liver cell types (NPCs incl. Kupffer cells and liver endothelial cells)^18^. For the HeCaToS project, all hepatic microtissues were generated from the same base materials: a multidonor batch of primary human hepatocytes (5 males and 5 females, aged between 7–59 years) and NPCs from a single Caucasian 27-year-old person of unreported gender (Cat # MT-02-302-04). These microtissues were cultured in 3D InSight^TM^ Human Liver Microtissue Maintenance Medium-AF (Cat #CS-07-001a-01) containing galactose to facilitate examination of mitochondrial effects.

The 3D InSight^TM^ Human Cardiac Microtissue model was supplied for beta-testing and is not commercially available^5^. It consists of approximately 4000 induced pluripotent stem cell (iPSC)-derived human cardiomyocytes (female donor, no disease phenotype) in co-culture with approximately 1000 cardiac fibroblasts (Caucasian male, 18 years old). These microtissues were cultured in 3D InSight^TM^ Human Cardiac Microtissue Maintenance Medium (Cat#CS-07-010-01) containing galactose to facilitate examination of mitochondrial effects.

### PBPK modeling & exposure

For each compound, a PBPK model was applied to predict the *in vivo* compound exposure in either the liver (for hepatotoxicants) or the interstitial space of the heart (for cardiotoxicants)^7^. The models were designed to resemble the repetitive administration (1x daily) of either a therapeutic or a toxic dose of the compound for a duration of 2 weeks. The therapeutic dose was based on a standard clinical dosing scheme. The toxic dose was dependent on *in vitro* viability tests, from which the IC20 after 7 days was converted to the toxic dose through reverse dosimetry. The open source PK-sim software was used to create the models following a previously described workflow^7^, which were validated according to best practice guidelines^19^.

The general experimental design per compound used 96 well plates of microtissues, which were generated from the same starting material. The continuous dosing profiles obtained from the PBPK-modeling were implemented within an experimental setting through three medium changes per workday^2^, in which a compound stock solution (dissolved in DMSO) and DMSO solution (to a final concentration of 0.01%) were added to the culture medium of the microtissues. This implied a high dose for 2 hours, a medium dose for 6 hours and a low dose for the remaining 16 hours. For practical reasons, the medium was not changed during weekends, which always occurred from 72h-144h and 240h-312h. For the weekends dosing, an average concentration was calculated by the PBPK-expert team. The microtissues were exposed to this weekend dose for 72 hours. The dosing profiles of Azathioprine were included in Fig. 1 as an example.

The exact dosing profiles for each compound can be found on BioStudies, under accession number S-HECA8, Sample list.xlsx. Furthermore, all PBPK models developed in HeCaToS have been established in the PBPK software tool PK-Sim which is provided as part of the Open Systems Pharmacology platform (http://www.open-systems-pharmacology.org/). The models developed within HeCaToS are hence freely accessible and usable by the scientific community.

### Patient biopsies (cardiac)

Cardiac biopsies were obtained from patients (n = 14) with decreased left ventricular function. All patients that underwent endomyocardial biopsies (EMB) first had a physical examination, blood sampling, 12-lead electrocardiogram, 24-h Holter monitoring on indication, and a complete echocardiographic and Doppler evaluation. Significant coronary artery disease as a cause of the decreased ejection fraction was excluded by a coronary angiography (CAG) or a CT-angiography at baseline. EMB were performed as part of routine diagnostic work-up in nonischemic, non-valvular cardiomyopathy, upon consent of the patient, as part of the Maastricht Cardiomyopathy Registry with inclusion and exclusion criteria as described previously^20^. The main indication for EMB was a left ventricle ejection fraction (LVEF) <45% after 6 months of optimal medical treatment, and the absence of other.

Patients could be divided in two groups: 1) dilated cardiomyopathy ((DCM defined as LVEF <50% with an indexed left ventricular end diastolic diameter (LVEDDi) >33 mm/m2 (men) or >32 mm/m2 (women) measured by echocardiography), and 2) non-dilated cardiomyopathy (HNDC defined as LVEF <50% with an LVEDDi ≤33 mm/ m2 (men) or ≤32 mm/m2 (women) measured by echocardiography in the absence of a (i) myocardial infarction and/or significant coronary artery disease; (ii) primary valvular disease; (iii) hypertensive or congenital heart disease; (iv) acute myocarditis; (v) arrhythmogenic right ventricular dysplasia; and (vi) hypertrophic, restrictive or peripartum cardiomyopathy). For the HeCaToS project, we included cases with a previous history of anthracycline chemotherapy, and control DCM/HNDC without this treatment. Disease and control patients were matched based on age, gender, BMI, and LVEF. The study was performed according to the declaration of Helsinki and was approved by the Medical Ethics Committee of Maastricht University Medical Centre. All patients gave written informed consent.

Data and detailed patient information can be found on BioStudies, under accession number S-HECA35 (proteome), S-HECA469 (transcriptome) and S-HECA510 (miRNA profiles).

### Patient serum samples (hepatic)

Serum samples from drug induced liver injury (DILI) patients with different phenotypes over time were obtained through an observational longitudinal clinical study. Written informed consent to participate in the study was obtained from 79 patients that underwent DILI evaluation at the Clinical Hepatotoxicity Unit between 2013 and 2018. These serum samples were used for metabolic analysis, which has already been fully described by Quintás *et al*.^11^.

### Isolation of DNA, RNA, protein (patient biopsies)

Tissue disruption of the patient specimen was done by cryogenic grinding (mortar and pestle in liquid nitrogen). Thereafter, the Trizol/Qiazol protocol (Qiagen, Cat #79306) was applied for RNA isolation^21^. Concentrations of RNA were measured with Qubit 2.0 Fluorometer system (Thermo Fisher Scientific, Waltham, MA USA) and the RNA quality was assessed using an Agilent 2100 Bio-analyzer (Agilent Technologies, Palo Alto, CA). For proteomics analysis, the isolation method was identical to the one applied for the *in vitro* samples (method described below).

### Isolation of DNA, RNA, protein, metabolites (*in vitro*)

The two-week exposure *in vitro* included 7 sampling time points per dosing profile, with 3 replicates per time point. To obtain sufficient material for analysis techniques, each replicate consisted of multiple microtissues (36 cardiac or 54 hepatic), which were individually exposed and thereafter pooled before isolation with the AllPrep DNA/RNA/miRNA Universal Kit (Qiagen, Cat #80224). Furthermore, proteins were extracted from a pool of 18 microtissues (incubated individually and in parallel with microtissues used for DNA/RNA) using freeze-thaw cycles followed by centrifugation as described previously^2^. Finally, the medium of the exposed microtissues was used for metabolomics analysis. For metabolomics analysis, 2 additional sampling time points were used: 144 h and 312 h. Because these samples were obtained after a weekend exposure, in which no medium changes took place, metabolites accumulated over 72 h thus facilitating their detection. Batches for data generation (epigenomics, transcriptomics, proteomics and metabolomics) contained all samples that were obtained during an exposure run and therefore correspond to the exposure date (start) included in Appendix I.

### Epigenomics/MeDIP-seq data analysis

A modified version^2^ of the low input MeDIP protocol^22^ was used to prepare MeDIP-Libraries. In short, the Covaris S2 system was used to obtain DNA fragments of 100–200 bp. The NEBNext® Ultra™ library prep kit for Illumina® (NEB) was used to perform end repair and A tailing, followed by adapter ligation with NEBNext® Ultra™ Ligation Module (NEB). Samples were purified using Agencourt® AMPure® XP beads (Beckman Coulter) and the MagMeDIP kit (Diagenode) was used to capture Methylated fragments. Library concentration was determined by Qubit™ and qPCR and quality of the libraries was assessed on an Agilent Bioanalyzer 2100. To gain exhaustive genome-wide coverage for MeDIP-seq data analysis, the triplicate samples that have been sequenced individually, were merged before alignment. MeDIP sequencing reads were aligned to the GRCh38 reference genome using bwa Version 0.7.15-r1140^23^, and analyzed in 250 bp windows using the R/ Bioconductor package QSEA^24^ with standard parameters. Within QSEA, the MeDIP enrichment was calibrated using 450k methylation array measurements of primary hepatocytes (GSM999339) and cardiac myocytes (HCM, GSM999381) from ENCODE^25^, for the hepatic and cardiac microtissues, respectively. To this end, beta values of the calibration samples were computed by means of the R/Bioconductor package Minfi^26^, genomic locations of the array probes were mapped from GRCh37 to GRCh38 using the UCSC liftOver command line tool^27^, and probes within 250 base windows were averaged. Differentially methylated regions obtained from QSEA were annotated using gene, exon, and promoter (transcription start site ± 2 kilobases) information from RefSeq, ENCODE TFBS and model-based CpG islands, all obtained via the UCSC table browser. Since ENCODE TFBS were not available for GRCh38, genomic locations were mapped from GRCh37 using the liftOver tool.

### Total RNA sequencing

All sequenced RNA samples had RNA integrity number (RIN) values above 6. Ribosomal depletion of the isolated total RNA was accomplished using the Illumina RiboZero Gold kit rRNA Removal kit (Cat #MRZG12324). Thereafter, sequencing libraries were prepared using the Lexogen SENSE total RNA library preparation kit (Cat #009.96). The quality of the library preparation was assessed using the Agilent 4200 TapeStation and the library concentration was determined using Qubit^TM^. The libraries were sequenced on the Illumina HiSeq 2500 (100 bp paired-end). After quality control of the data using FastQC^28^, trimmed reads were mapped to the human genome version hg38 and annotated with the GENCODE release 26 annotation. Reads were mapped using the splice junction mapper STAR^29^ and quantified using an algorithm based on Cufflinks^30^. Features used for quantification were the protein coding and the non-protein coding sequences (pseudo-genes missing a CDS of the transcripts). Differential expression analysis of the RNA-seq experiments was performed by means of DESeq 2^31^ (differential gene expression analysis based on the negative binomial distribution), an analysis tool suitable to detect differences in the raw read counts of features between two or more experiment groups.

### MicroRNA sequencing

An aliquot of the isolated total RNA (see above) was used for size selection and ligation using the TruSeq Small RNA Library Prep Kit (Illumina®, Cat #RS-200-0012, RS-200-0024, RS-200-0036). Thereafter, libraries were sequenced on the HiSeq 2500 in single-end mode and adapter sequences were removed and the resulting reads of 16–35 bp in length were aligned to the human genome using PatMaN^32^. No gaps or mismatches were allowed during this alignment. The mapping output was parsed (using the script in Appendix II) to obtain complete read counts for 3′ and 5′ miRNA species that were used to investigate differential expression using DESeq 2^31^.

### Proteomics

Peptides were measured on an Orbitrap Fusion mass spectrometer (Thermo Fisher Scientific) coupled to either 1) NanoLC-2D HPLC system (Eksigent, Dublin, CA) with an Easy-Spray Column (75 μm × 500 mm) packed with reverse-phase C18 material (Silica 100 Å, 2 μm), or 2) EASY-nLC 1000 system (Thermo Fisher Scientific, Germany) with a self-made column (75 μm × 150 mm) packed with reverse-phase C18 material (ReproSil-Pur 120 C18-AQ, 1.9 μm, Dr. Maisch HPLC GmbH). Peptides were loaded onto the column from a cooled (4 °C) Eksigent autosampler and separated with a linear gradient of acetonitrile/water, containing 0.1% formic acid, at a flow rate of 300 nl/min. A gradient from 5 to 30% acetonitrile in 60 minutes was used. The mass spectrometer was set to acquire full-scan MS spectra (300–1500 m/z) at 120,000 resolution at 200 m/z; precursor automated gain control (AGC) target was set to 400,000. Charge-state screening was enabled, and precursors with +2 to +7 charge states and intensities >5,000 were selected for tandem mass spectrometry (MS/MS). Ions were isolated using the quadrupole mass filter with a 1.6 m/z isolation window. Wide quadrupole isolation was used, and injection time was set to 50 ms. The AGC values for MS/MS analysis were set to 5,000 and the maximum injection time was 300 ms. HCD fragmentations were performed at a normalized collision energy (NCE) of 35%. MS/MS spectra were detected in the ion trap in 3 centroid mode. Precursor masses previously selected for MS/MS measurement, were excluded from further selection for 25 s, and the exclusion window was set at 10 ppm. Raw data were pre-processed in the Genedata Expressionist® software using a classical bottom-up LC-MS proteomics workflow and included the following major blocks of activities: pre-processing of raw data, peak detection and isotope clustering, identification and validation of peptides/proteins, preliminary statistics and data evaluation, export and reporting of processed data. After the data pre-processing, the intensities were log2 transformed, normalized, and 2-sided T-tests were then used for the determination of differentially expressed proteins (DEPs) in comparison with the corresponding time-matched controls.

### Metabolomics

Multiple metabolomics methods were applied to *in vitro* exposed samples. To generate lipid extracts, 48 pooled tissues of the 72 h time point were subject to a modified Bligh-Dyer procedure (3:2:1 H_2_O:CHCl3:MeOH) and the organic fraction isolated. Lipidomic profiles were generated using LC-MS/MS methodologies. To detect changes in the acylcarnitine, lysophospholipid and bile acid reversed phase (RP) methodology, more specifically an RP UPLC-QToF-MS method^33^ was used. Polar metabolite profiles (including amino acids, nucleotides and organic acids) were analyzed using hydrophilic interaction liquid chromatography (HILIC) methodology. The QTOF method was also applied to pooled culture media of replicated wells for each time point. The analysis workflow^11^ also included: randomized injections, blank signals subtraction, signal deconvolution and instrumental batch correction. Finally, to quantify levels of small metabolites, global hydrogen-1 nuclear magnetic resonance (^1^H-NMR) spectroscopy was used to generate metabolic profiles. Samples from all time points were included to facilitate the detection of changes over time. The mass spectrometry data was processed with XCMS^34^. Peak detection was done using the *centWave* method, the *wMean* function was used to calculate the intensity weighed *m/z* values of each feature, peak matching across samples was performed using the *nearest* method and the *fillPeaks* method was used to fill missing data points. Comparison of automated and manual integration results of endogenous metabolites and internal standards was done to assess peak integration and alignment accuracies. Within batch effect correction and between-batch effect correction was carried out as described by Kuligowski *et al*.^35^ and Sánchez-Illana *et al*.^36^. Metabolite annotation was done as described by Ten-Doménech *et al*.^37^ using the Human Metabolome Database (http://www.hmdb.ca), METLIN databases (http://www.metlin.scripps.edu), and LipiDex^38^. Metabolite classes and subclasses were obtained from the Human Metabolome Database and incorporated automatically in the annotation process. T-tests with FDR-adjusted p values were used to determine significant metabolite changes.

### Functional assays

ATP content of the microtissues was measured using Promega’s CellTiter Glo 3D (Cat #G9683) according to manufacturer’s protocol, in which the microtissues were incubated for 30 min with luciferase reagent and the luminescence was measured.

Apoptosis induction was measured using Promega’s caspase-Glo® 3/7 assay (Cat #G8092) according to manufacturer’s protocol. Briefly, the Z-DEVD-aminoluciferin substrate is cleaved by caspase 3/7, releasing a substrate for luciferase (aminoluciferin), resulting in measurable luminescence.

Mitochondrial function after 2 h and 7 days of DOX treatment was assessed by measuring extracellular oxygen consumption using Luxcel’s MitoXpress® Xtra Oxygen Consumption Assay (Cat #MX-200) according to manufacturer’s protocol. In short, oxygen quenches the MitoXpress® Xtra probe, making the measured fluorescent signal, inversely proportional to the oxygen concentration.

## Data Records

The HeCaToS data collection thus contains datasets for multi-omics responses to 10 hepatotoxic compounds, 10 cardiotoxic compounds and corresponding controls. For each compound, measurements were obtained for two dosing profiles (therapeutic and toxic) at 7 time points (2-8-24-72-168-240-336 h for therapeutic profile; 0-2-8-24-72-168-240 h for toxic profile), which were measured with three replicates. Therefore, a compound dataset comprised of (2*7*3 = ) 42 samples and a control dataset contained either 21 samples (7*3) or 24 samples (8*3), depending on whether the T0 time point was included (see usage note for details). In total, 990 *in vitro* samples were generated, of which 474 hepatic and 516 cardiac. Furthermore, we also obtained 51 human cardiac biopsies for validation of *in vitro* observations.

Massive amounts of data were obtained using high-throughput omics technologies, in total 8.67 TB. MeDIP-seq was used for the assessment of methylation changes. 1124 files were generated with a total size of 1698.2 GB. Transcriptomics data included both RNA expression (RNA-seq) and microRNA profiles (miRNA-seq). RNA-sequencing (depleted of ribosomal RNAs) resulted in 1069 paired-end libraries (2138 files) with a combined size of 5570.3 GB. Single-end miRNA libraries resulted in 316.2GB of data stored in 903 files. Proteomics included 1100 LC-MS/MS mass spectrometry raw data sets with a total volume of 1089.1 GB. And finally, metabolomics technology generated 4.0 GB of data obtained from 673 ^1^H-NMR spectra, 165 HILIC LC-MS/MS chromatograms, 210 RP LC-MS/MS chromatograms and 184 LC-MS QTOF directories.

All generated data within the HeCaToS project are grouped into datasets with unique accession numbers. To increase the findability of a specific dataset, the accession number of a specific dataset can be easily obtained from Appendix I.

With the rapid evolution of bioinformatics tools, we recommend all future users of the HeCaToS data to:

1. Start from the supplied raw data files;
2. Make use of updated annotation information for genes, proteins and/or metabolites; and 3) process the data by means of the latest bioinformatics tools.

Despite this recommendation, the BioStudies data repository does contain processed transcriptomics and proteomics datasets used by the HeCaToS consortium. In summary, the following data analyzes were performed:

1. Normalized expressed genes or detected proteins were compared for each time point with the corresponding time-matched control. These analyzes reflect the effect of the applied compounds in comparison with ‘untreated’ microtissues;
2. A two-step regression procedure was applied to identify targets with significantly different time-dependent expression profiles of the treatment/dose experiments in comparison with the control experiments. Rather than comparing single time point experiments with the corresponding controls, this analysis focused on differences in the time-course profiles;
3. Normalized expressed genes or detected proteins of each time point of a treatment/dose group was compared with the time point 0 hr (T0). Such analyzes provide information about time-dependent expression changes independent of the controls without considering the whole time course profile.

## Technical Validation

Quality assessment of the generated datasets within the HeCaToS project revealed high quality of most samples, including patient samples. Due to the large amount of data, the quality reports were included as supplementary data. To give a general impression of the data quality, the main text does include an overview of the coverage and quality score for the three most data-rich omics technologies: epigenomics, transcriptomics and proteomics.

Multiple omics technologies applied within the HeCaToS project were based on sequencing technology that resulted in data in fastq format. Therefore, the epigenome data (MeDIP-seq) and transcriptome data (RNA-seq) were processed using the same quality control pipeline. A very important step in analyzing sequencing data is the removal of technical adapters, including barcodes, which are necessary for obtaining the data but hamper downstream analysis. The Fastp tool^39^ was used for simultaneous adapters removal, quality filtering and quality control. Thereafter, the MultiQC tool^40^ was used to generate a single QC report for all the analyzed samples, which were included as supplementary data. These reports contain all QC-metrics assessed by Fastp, including sequencing depth, Q30 quality scores, filtered reads classification, duplication rates, insert sizes, GC content and N content.

**Fig. 2:**
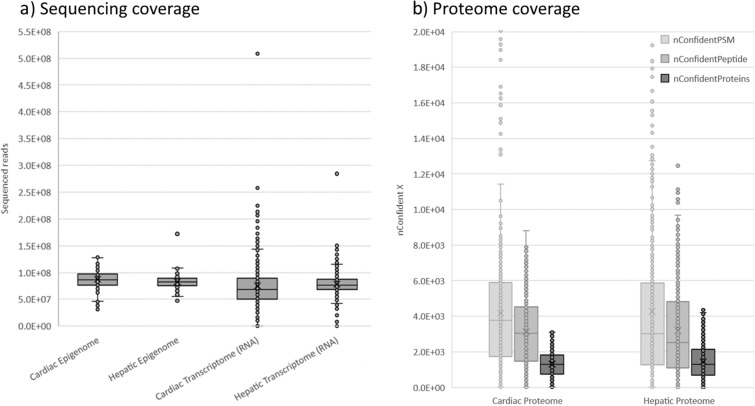
Coverage of generated omics datasets. (**a**) Shows the sequencing coverage of epigenomic and transcriptomic data. Each data point represents a single sample for which the amount of raw sequencing reads was depicted. (**b**) Shows the coverage of proteomic data through the amount of confident PSM, confident peptides and confident proteins. Each data point represents a single sample.

**Fig. 3:**
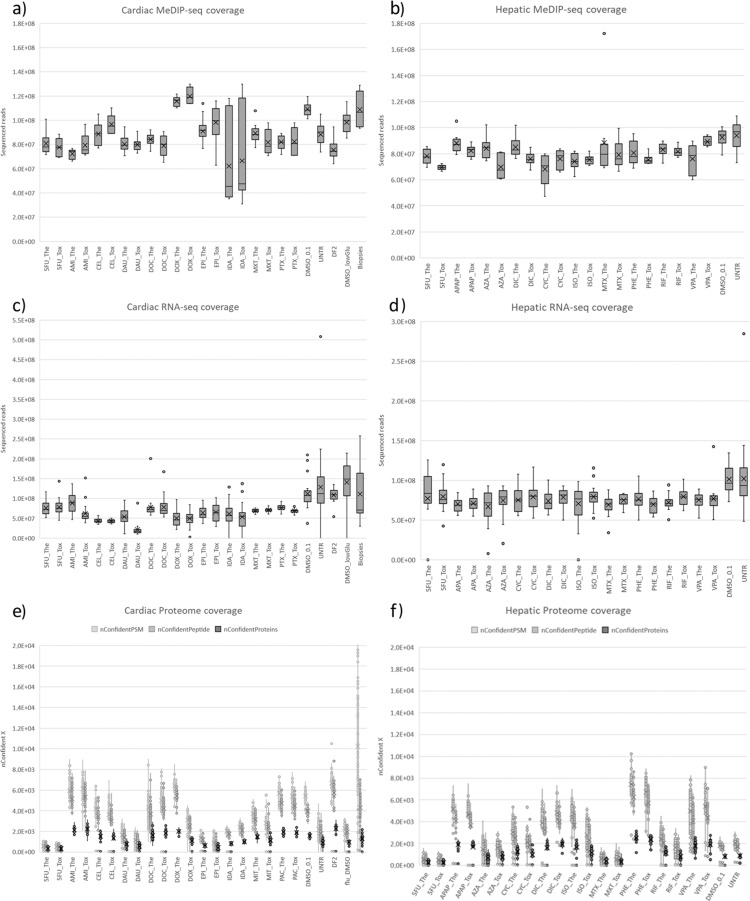
Coverage of individual omics datasets. (**a,c,e**) Contain cardiac datasets, while (**b,d,f**) contain hepatic datasets. (**a,b**) Depicts sequencing coverage of epigenomic data, and (**c,d**) sequencing coverage of transcriptomic data. For these sequencing technologies, raw sequencing reads were depicted for each measured sample. (**e,f**) Depicts coverage of proteomic data for each measured sample through the amount of confident PSM, confident peptides and confident proteins.

The most important QC-metrics for sequencing data are sequencing depth (also referred to as coverage) and Q30 quality scores. An overview of the sequencing depth is included in Fig. 2a, where datasets are grouped based on tissue type. A more detailed view is included in Fig. 3a, in which each dataset (aka. compound) is depicted separately. The sequencing depth of the epigenome ranged from 31 M to 172 M reads, with an average of 83 M (sd: 13 M) reads for hepatic samples and 87 M (sd: 17 M) reads for cardiac samples. The quality of the reads was excellent, on average 96.5% (sd: 1.2%) of reads were above Q30. The RNA sequencing data exhibited a greater variance, with a coverage ranging from 19 M to 508 M and an average of 79 M (sd:22 M) and 77 M (sd:43 M) for hepatic and cardiac samples respectively. We advise future users to discard samples with coverage below 20 M (6 hepatic and 24 cardiac *in vitro* samples). Sequencing quality was good for all samples with on average 93.4% (sd: 5.5%) of reads above Q30.

Quality control report of proteomics spectra (see supplementary data) was generated using a custom R Markdown script (Appendix III). The protein mass spectrometry search engine Comet^41^ was used to assess the quality of the raw proteomics files. Comet uses the Mascot Generic Format (MGF) files as input, generated from the raw file using the R package rawrr^42^. Default settings were used for low-resolution HCD MS2 spectra, with fixed modification Carbamidomethyl(C) and variable modification Ox(M). Comet reports a score for each peptide spectrum match (PSM). Using the target decoy search results, the false discovery rate (FDR) for each PSM was determined, using the functions implemented in the R package protViz^43^. The peptide and protein FDR were computed after filtering the PSMs for an FDR of 1%.

An overview of the numbers of confident PSMs (nConfidentPSM), Peptides (nConfidentPeptide), and Proteins (nConfidentProteins) are included in Fig. 2b, in which datasets are grouped based on the tissue type. A more detailed view is included in Fig. 3b, in which each compound dataset is presented separately. The coverage for proteomics data can be assessed using the number of confident proteins (nConfidentProteins) QC metric. An average of 1447.88 (sd:992) and 1304.0 (sd:697.4) confident proteins were identified for hepatic and cardiac samples. Furthermore, the assignment Rate (assignmentRate), which describes the ratio of nConfidentPSM/nPSM, ranged between 0–26 with an average of 7.5 (sd:5.4).

## Usage Notes

As stated earlier, we recommend all future users of the HeCaToS data to 1) start from the supplied raw data files; 2) make use of updated annotation information for genes, proteins and/or metabolites; and 3) process the data with the latest bioinformatics tools.

Due to the multi-omics measurements gained from the same or identical *in vitro* exposures, the HeCaToS data constitutes a data-goldmine that can be used for a wide variety of applications. The data can be used to broaden our understanding of toxicity mechanisms through cross omics analysis, investigate gene regulation networks or assess time- and dose dependent toxic effects. It may serve as a source to generate, expand or validate adverse outcome pathways (AOPs). It can be used as input to improve bioinformatic tools and pipelines or to create entirely new ones. Furthermore, the RNA-sequencing data can be used to investigate novel targets such as circular RNAs or long non-coding RNAs. The data can also be applied to benefit global health through the identification of biomarkers for diagnosis of toxic exposures or to inform and validate computational models for risk prediction.

As in any project, not everything went as foreseen. We did encounter some minor obstacles that we had to overcome. To make sure that the data can be used to its fullest potential, we list below the main points, that may have an impact on the way the data is to be used.

1. PBPK-models resembled repetitive administration (1x daily) of a compound, which is realistic for many of the investigated compounds. However, also for chemotherapeutics the model administered a therapeutic (or toxic) dose 1x daily, while in clinical practice there are weeks between the repetitive doses.
2. Exposures involved medium changes 3x per day on weekdays, with doses specified by the PBPK model. On weekends, the medium was not changed. Instead of the three different doses per day, a weekend average was calculated by the PBPK-expert team. Weekends always occurred from 72h-144h and 240h-312h of the exposure.
3. Most toxic doses were based on IC20 observed in the microtissues after 7 days of drug treatment. However, for some compounds, this caused solubility problems. In these cases, the maximum dose was obtained at maximum solubility. Consequently, for some compounds, the therapeutic dose and toxic dose were not executed on the same day. This occurred for exposure to Cyclosporin A, Isoniazid and Valproic acid in hepatic microtissues and exposure of Amiodarone and Docetaxel in cardiac microtissues.
4. Since all compounds in the HeCaToS project were dissolved in 0.1% DMSO, a vehicle control of 0.1% DMSO was created. However, since the exposures mimicked *in vivo* drug concentrations through three medium changes with PBPK-calculated concentrations, the amount of added drug, and thus the amount of DMSO, differed per time point. This issue was noticed after completion of the anthracyclines Idarubicin, Doxorubicin, Epirubicin and Daunorubicin datasets. To make use of this data, we generated a vehicle control with the same fluctuating DMSO concentrations, which we named fluctuating control II (DF2). For all other compounds, we adjusted the DMSO in the exposed microtissues to 0.1% to be in concordance with the 0.1% DMSO vehicle control.
5. The two-week exposure included 7 sampling time points per dosing profile. Initially it was planned to sample at 2, 8, 24, 72, 168, 240 and 336 h for both doses. Because the toxic 336 h exposure resulted in low DNA and RNA yields due to toxicity, this time point was replaced with a baseline measurement (T0 control sample) for therapeutic and toxic samples. T0 control samples are available for all datasets except for the initial exposures (Ida, Dox, Epi and the cardiac 0.1% DMSO control).
6. Most compounds contain their own T0 control samples. However, compound exposures run at the same time from the same batch of microtissues, share common T0 samples. To ease data analysis, we included the T0 samples with both compounds. Therefore, some unique sample numbers were duplicated in Appendix I.
7. It has to be kept in mind that control samples (DMSO & UNTR) were run in a separate batch. It is therefore important to think about possible batch effects and correct for them (e.g. use the T0 samples for batch correction) or formulate research questions that avoid batch effects (e.g. perform comparative analysis between therapeutic and toxic doses).
8. DNA yields were lower than expected for the first epigenetics datasets (Doxorubicin and the 0.1%DMSO vehicle control). To perform MeDIP analysis, the three replicates of each time point were pooled together. For all other datasets, DNA yields were improved by optimizing the DNA extraction protocol and measured in triplicate.
9. Epigenomics data was obtained for all time points for the cardiotoxic anthracyclines (Dox, Epi, Ida and Dau). For other compounds, three time points were selected (0, 72 and 168 h).
10. A major advantage of the HeCaToS dataset is that multi-omics data was obtained from the same microtissues. The only exception is Idarubicin (proteomics samples failed in the lab, redone on a new batch of microtissues).
11. The HeCaToS data collection contains two entries for Acetaminophen (APAP) and Azathioprine (AZA). The first datasets were obtained very early in the project, before we noticed the fluctuating of DMSO (as described in usage note 4). We redid the exposures with adjusted DMSO concentrations. Therefore, these datasets were named APAP(II) and AZA(II).
12. Total RNA datasets were obtained from ribo-depleted samples. Unfortunately, the library prep for hepatic 5FU failed. With the leftover RNA, we were still able to perform mRNA sequencing for this compound.

## Supplementary information

Appendix I

Appendix II

Appendix III

## Data Availability

Since bioinformatics tools and pipelines are continuously updated, we urge researchers to start their analysis from the raw data and use up to date bioinformatics approaches. Therefore, we did not supply the specific code used during the HeCaToS project. Appendix II & III contain custom R-scripts for microRNA parsing and proteomics QC respectively.
