# Supplementary material for: Multi-omics HeCaToS dataset of repeated dose toxicity for cardiotoxic & hepatotoxic compounds
Source: Sci Data. 2022 Nov 14;9:699. doi: 10.1038/s41597-022-01825-1 (PMC9663581; doi:10.1038/s41597-022-01825-1)
Supplement: Supplementary file 2 — Appendix II [file 41597_2022_1825_MOESM2_ESM.docx]

#Appendix II: Script for MicroRNA_Parsing

# Parse the output of patman (pat files) into a more usefull format

predir <- "set/the/directory/containing/the/pat/files/"

WORK.DIR <- paste(predir)

setwd(WORK.DIR)

patt <- "*pat"

fichier <- list.files(pattern = patt)

taille <- length(fichier)

for (i in 1:taille) {

temp1 <- read.table(fichier[i], header = FALSE, stringsAsFactors=FALSE, sep = "\t")

temp1 <- as.data.frame(sapply(temp1,gsub,pattern=')',replacement=""))

temp1 <- as.data.frame(sapply(temp1,gsub,pattern="\\(",replacement=" "))

temp1 <- as.data.frame(sapply(temp1,gsub,pattern=" ",replacement="\t"))

fileoutput= paste(strsplit(fichier[i],split=".fastq")[[1]][1],".out",sep="")

write.table(temp1, file = fileoutput, col.names=FALSE, row.names = FALSE, quote = FALSE, sep = "\t")

}

rm(temp1)

## Parse the out files to :

### Divide the count for each sequence base on the hits number for this sequence

### Rename the mature 3p and 5p depending on there position

### Create two files : mature and isomirs count.

patt <- "*out"

fichier <- list.files(pattern = patt)

taille <- length(fichier)

for (i in 1:taille) {

temp2<- read.table(file =fichier[i], header = FALSE, stringsAsFactors=FALSE, sep = "\t")

colnames(temp2)<-c("mature","mirbase", "pre_size","sequence","count","start","end","strand","mismatch")

print(paste("processed file :", fichier[i]))

for (j in 1:nrow(temp2)) {

## Divide the count number by the number of occurence of this sequence in the mapping

temp2$count[j] <- temp2$count[j]/nrow(subset(temp2,temp2$seq == temp2$seq[j]))

## assign 5p or 3p depending on the mature position in the pre-miR

if (temp2$start[j] <= temp2$pre_size[j]/2) {

temp2$mature[j] <- paste(temp2$mature[j],"_5p",sep="")

}

else {

temp2$mature[j] <- paste(temp2$mature[j],"_3p",sep="")

}

}

fileoutput= paste(strsplit(fichier[i],split=".out")[[1]][1],".isomirs",sep="")

write.table(temp2, file = fileoutput, row.names = FALSE, quote = FALSE, sep = "\t")

mature_count <- rowsum(temp2$count,temp2$mature, reorder=FALSE)

fileoutput= paste(strsplit(fichier[i],split=".out")[[1]][1],".mature",sep="")

write.table(mature_count, file = fileoutput, row.names = TRUE, col.names=FALSE, quote = FALSE, sep = "\t")

}

rm(temp2)

#### Merge the mature files into one Raw count per sample

compounds <- c("Compound1","Compound2","Compound3")

for (x in 1:length(compounds)) {

merge_data <- NULL

patt <- paste(compounds[x],".*mature",sep="")

fichier <- list.files(pattern = patt)

taille <- length(fichier)

temp1<- read.table(file =fichier[1], header = FALSE, row.names=1, sep = "\t")

colnames(temp1)<-strsplit(fichier[1],split=".mature")[[1]][1]

temp2<- read.table(file =fichier[2], header = FALSE, row.names=1, sep = "\t")

colnames(temp2)<-strsplit(fichier[2],split=".mature")[[1]][1]

merge_data<- merge(temp1,temp2,by="row.names", all=TRUE)

for (i in 3:taille) {

temp1 <- read.table(file =fichier[i], header = FALSE, row.names=1, sep = "\t")

print(paste("Processed files: ",fichier[i],sep=""))

colnames(temp1)<-strsplit(fichier[i],split=".mature")[[1]][1]

merge_data <- merge(merge_data,temp1,by.x="Row.names",by.y="row.names",all=TRUE)

}

# merge_data[is.na(merge_data)]<-0

write.table(merge_data, file =paste(compounds[x],"mature_counts.txt",sep="_"), row.names = FALSE, quote = FALSE, sep = "\t")

}

#### Merge the mature files into one Raw count per sample

for (x in 1:length(compounds)) {

merge_data <- NULL

patt <- paste(compounds[x],".*isomirs_5p",sep="")

fichier <- list.files(pattern = patt)

taille <- length(fichier)

temp1<- read.table(file =fichier[1], header = FALSE, row.names=1, sep = "\t")

colnames(temp1)<-strsplit(fichier[1],split=".isomirs")[[1]][1]

temp2<- read.table(file =fichier[2], header = FALSE, row.names=1, sep = "\t")

colnames(temp2)<-strsplit(fichier[2],split="isomirs")[[1]][1]

merge_data<- merge(temp1,temp2,by="row.names", all=TRUE)

for (i in 3:taille) {

temp1 <- read.table(file =fichier[i], header = FALSE, row.names=1, sep = "\t")

print(paste("Processed files: ",fichier[i],sep=""))

colnames(temp1)<-strsplit(fichier[i],split=".isomirs")[[1]][1]

merge_data <- merge(merge_data,temp1,by.x="Row.names",by.y="row.names",all=TRUE)

}

# merge_data[is.na(merge_data)]<-0

write.table(merge_data, file =paste(compounds[x],"iso5p_counts.txt",sep="_"), row.names = FALSE, quote = FALSE, sep = "\t")

}

## change the id of isomirs per sample

patt <- "*isomirs"

fichier <- list.files(pattern = patt)

taille <- length(fichier)

for (i in 1:taille) {

iso_data<- read.table(file =fichier[i], header = TRUE, stringsAsFactors=FALSE, sep = "\t")

for (j in 1:nrow(iso_data)) {

## assign 5p or 3p depending on the mature position in the pre-miR

iso_data$mature[j] <- paste(iso_data$mature[j],iso_data$start[j],iso_data$strand[j], sep="_")

}

print(fichier[i])

iso5p_count <- rowsum(iso_data$count,iso_data$mature, reorder=FALSE)

write.table(iso5p_count, file = paste(fichier[i],"_5p",sep=""), row.names = TRUE, col.names=FALSE, quote = FALSE, sep = "\t")

}

for (i in 1:taille) {

iso_data<- read.table(file =fichier[i], header = TRUE, stringsAsFactors=FALSE, sep = "\t")

for (j in 1:nrow(iso_data)) {

## assign 5p or 3p depending on the mature position in the pre-miR

iso_data$mature[j] <- paste(iso_data$mature[j],iso_data$start[j],iso_data$end[j],iso_data$strand[j], sep="_")

}

print(fichier[i])

write.table(iso_data, file = fichier[i], row.names = FALSE, col.names=FALSE, quote = FALSE, sep = "\t")

}

#### Merge the isomRs files into one Raw count per sample

for (x in 1:length(compounds)) {

merge_data <- NULL

patt <- paste(compounds[x],".*isomirs",sep="")

fichier <- list.files(pattern = patt)

taille <- length(fichier)

temp1<- read.table(file =fichier[1], header = FALSE, sep = "\t", stringsAsFactors= FALSE )

temp1 <- cbind(temp1[,1],temp1[,5])

colnames(temp1)<-c("iso_id",strsplit(fichier[1],split=".isomirs")[[1]][1])

temp2<- read.table(file =fichier[2], header = FALSE, sep = "\t", stringsAsFactors= FALSE )

temp2 <- cbind(temp2[,1],temp2[,5])

colnames(temp2)<-c("iso_id",strsplit(fichier[2],split=".isomirs")[[1]][1])

merge_data<- merge(temp1,temp2,by="iso_id", all=TRUE)

for (i in 3:taille) {

temp1 <- read.table(file =fichier[i], header = FALSE, sep = "\t", stringsAsFactors= FALSE)

print(paste("Processed files: ",fichier[i],sep=""))

temp1 <- cbind(temp1[,1],temp1[,5])

colnames(temp1)<-c("iso_id",strsplit(fichier[i],split=".isomirs")[[1]][1])

merge_data <- merge(merge_data,temp1,by="iso_id", all=TRUE)

}

# merge_data[is.na(merge_data)]<-0

write.table(merge_data, file =paste(compounds[x],"isomirs_counts.txt",sep="_"), row.names = FALSE, quote = FALSE, sep = "\t")

}
