# Supplementary material for: Multi-omics HeCaToS dataset of repeated dose toxicity for cardiotoxic & hepatotoxic compounds
Source: Sci Data. 2022 Nov 14;9:699. doi: 10.1038/s41597-022-01825-1 (PMC9663581; doi:10.1038/s41597-022-01825-1)
Supplement: Supplementary file 3 — Appendix III [file 41597_2022_1825_MOESM3_ESM.docx]

#Appendix III: Script for Proteome QC with RMarkdown

---

title: "HeCaToS MS QC"

author:

- name: Christian Panse

affiliation:

- &fgcz Functional Genomics Center Zurich - Swiss Federal Institute of Technology in Zurich

- &sib Swiss Institute of Bioinformatics

- name: Witold E. Wolski

affiliation:

- *fgcz

- *sib

email: wew@fgcz.ethz.ch

bibliography: fgcz.bib

output:

BiocStyle::html_document

abstract: |

QC of all proteomics raw files made available on BioStudies by the Functional Genomics Center Zurich FGCZ.

vignette: |

%\VignetteIndexEntry{Vignette Title}

%\VignetteEncoding{UTF-8}

%\VignetteEngine{knitr::rmarkdown}

editor_options:

chunk_output_type: console

---

```{r setup, include=FALSE}

knitr::opts_chunk$set(echo = FALSE, message = FALSE)

```

# Introduction

To assess the quality of the raw files uploaded to BioStudies, we used the protein mass spectrometry search engine [@comet1,@comet2]. Comet uses the Mascot Generic Format (MGF) files as input, generated from the raw file using the R package _rawrr_ [@rawrr]. We are using the default settings for low-resolution HCD MS2 spectra, with fixed modification Carbamidomethyl(C) and variable modification Ox(M). Comet reports a score for each peptide spectrum match (PSM). Using the target decoy search results, we determined the false discovery rate (FDR) for each PSM, using the functions implemented in the R package _protViz_ [@protViz]. After filtering the PSMs for an FDR of 1%, we computed the peptide and protein FDR.

# Experiment Summaries

In Table 1, for each experiment we report the median of:

- nConfidentPSM

- nConfidentPeptide

- nConfidentProteins

- assignmentRate

- FDR_PSM

- FDR_Peptide

- FDR_protein

```{r read}

comet <- read.table("HeCaToS-comet.tsv", header = TRUE)

comet <- dplyr::select(comet, experiment, filename, instrument , assignmentRate , nPSM, nConfidentPSM, nConfidentPeptide,nConfidentProteins, fdrPSM , fdrPeptide, fdrProtein)

```

```{r nSample}

x.l <- aggregate(comet$nConfidentPeptide, FUN=length, by=list(comet$experiment))

colnames(x.l) <- c("Experiment", "nSamples")

```

```{r agg}

x <- aggregate(cbind(comet$nConfidentPSM, comet$nConfidentPeptide, comet$nConfidentProteins, comet$assignmentRate),

FUN=function(x){round(median(x))}, by=list(comet$experiment))

colnames(x) <- c("Experiment", "med_nConfidentPSM", "med_nConfidentPeptide", "med_nConfidentProteins", "med_assignmentRate")

xFDR <- aggregate( cbind( comet$fdrPSM * 100, comet$fdrPeptide*100 ,comet$fdrProtein*100) ,

FUN = function(x){round(median(x),digits=1)}, by=list(comet$experiment))

colnames(xFDR) <- c("Experiment", "med_FDR_PSM","med_FDR_Peptide","med_FDR_protein")

qcSummaries <- x.l |> merge( x) |> merge(xFDR)

SUMMARY <- list()

SUMMARY$experimentSummaries <- qcSummaries

```

```{r}

cap <- ""

knitr::kable(qcSummaries, caption = cap)

```

# File Summaries

Table 2 shows a QC summary for each raw file.

- experiment - name of the HeCaTos experiment

- filename - name of the raw file.

- instrument - instrument the data was aquired on

- assignmentRate - the assignment rate, which is the ratio of nConfidentPSM/nPSM

- nPSM - number of peptide spectrum matches

- nConfidentPSM - number of peptide spectrum matches with an FDR of less than 1%

- nConfidentPeptide - number of confidently identified peptides

- nConfidentProteins - number of confidently identified proteins

- fdrPSM - FDR on peptide spectrum match level

- fdrPeptide - FDR on peptide level

- fdrProtein - FDR on protein level

```{r cometData}

SUMMARY$fileSummaries <- comet

writexl::write_xlsx(SUMMARY, path = "QC_Summaries.xlsx")

comet |> knitr::kable()

```

# Side notes

This document was rendered by executing `R -e "rmarkdown::render('HeCaToS-MS.Rmd')"`.

The following software tools were used:

@comet1 @comet2 @protViz @rawrr

* `r Biocpkg("rawrr")`, _R_ packages available on _Bioconductor_ - mgf generation

* `r CRANpkg("protViz")`, _R_ packages available on CRAN - analysizing comet output

# Session info {.unnumbered}

```{r sessionInfo, echo=FALSE}

sessionInfo()

```

# References
